# Effects of Benzo(a)pyrene on the endometrial receptivity and embryo implantation in mice: An experimental study

**DOI:** 10.18502/ijrm.v16i12.3680

**Published:** 2019-01-28

**Authors:** Zeinab Mardanshahi, Abbasali Karimpour Malekshah, Fereshteh Talebpour Amiri, Reza Valadan

**Affiliations:** ^1^Department of Anatomy, Faculty of Medicine, Molecular and Cell Biology Research Center, Mazandaran University of Medical Sciences, Sari, Iran.; ^2^Student Research Committee, Faculty of Medicine, Mazandaran University of Medical Sciences, Sari, Iran.; ^3^Department of Immunology, Faculty of Medicine, Molecular and Cell Biology Research Center, Mazandaran University of Medical Sciences, Sari, Iran.

**Keywords:** *Benzo(a)pyrene*, * Embryo implantation*, * Estrogen, Progesterone*, * ALK5*, * E-cadherin.*

## Abstract

**Background:**

Benzo(a)pyrene (BaP) as an environmental pollutant is ubiquitous in the environment and it has destructive effects on human health. So far, various studies have demonstrated that BaP can cause adverse effects on the female reproductive system, but the existing information is limited about the effects of BaP on the endometrial receptivity and embryo implantation.

**Objective:**

The aim of this study was to investigate the effects of BaP on the endometrial receptivity and implantation in mice.

**Materials and Methods:**

In this experimental study, 40 pregnant BALB/c mice were divided into 5 groups (*n* = 8/each) as follows: experimental groups received the doses of 100 µg/kg, 200 µg/kg, and 500 µg/kg BaP dissolved in corn oil, the control group received normal saline and sham group received corn oil. Pregnant mice administered these solutions from Day 1 to Day 5 of gestation by gavage. On Day 6, the mice were sacrificed. Then their embryos were counted and the hormonal, histomorphological and molecular analyses were performed on the mocusa of uterine tube.

**Results:**

The data revealed that BaP reduces estrogen and progesterone levels, decreases the number of implantation site, endometrium thickness, uterine lumen diameter, stromal cells and endometrial glands, and blood vessels in the endometrium. However, the expression of Activin receptor-like kinase 5 and E-cadherin genes was not changed by BaP with different doses.

**Conclusion:**

The finding of this study showed that BaP can change estrogen and progesterone levels, and endometrial morphology leads to impairing the endometrial receptivity and decreasing the number of implantation site.

## 1. Introduction

Infertility is one of the significant medical complications today. The evidence indicates that infertility rate in human is declining (1). One of the most critical causes of human infertilities is the Endometrial Receptivity defect, resulting in implantation failure (2). The best endometrial condition for embryo implantation occurs within a limited time range, defined as the Implantation Window, which in human is from Day 20 to Day 24 of the normal cycle (3). In this period, some significant molecular and morphological changes occur in endometrium so that an appropriate environment is provided for the embryo attach, invasion, and penetration into the endometrium (4). These changes are associated with a set of molecular agents such as Cadherin proteins, transforming growth factor beta and hormonal agents like estrogen and progesterone, leading to embryo receptivity by endometrium (5).

Endometrial changes mainly occur by estrogen and progesterone. These hormones are critical for maintenance of the pregnancy (6). Steroids affect the regulating endometrial function and preparation for implantation. These hormones control cell functions such as proliferation, differentiation, and secretion paracrine or autocrine factors. Estrogen and progesterone cause some morphological changes in the endometrium including reduced mitotic activity, glandular secretion, thinning of the glycocalyx layer on the epithelial cells, stromal edema, and pinopodes during the menstrual cycle (5). E-cadherin plays an important role in the initial attachment of the embryo to the endometrium and implantation (7).

Moreover, it contributes to the important events of the embryonic development including gastrulation, neurulation, and organogenesis (6). Transforming growth factor beta also plays a significant role in different stages of reproduction including folliculogenesis, ovulation, decidua changes, implantation, and placenta formation (8–10). The signaling pathway of this protein involves ligand, receptor, and intermediate molecule, and impairment in each of these triple axes create the problem (9, 10). One of the most critical receptors of these proteins is Activin receptor like-kinase 5 (ALK5). Studies suggest that the absence of this receptor results in reduced fertility and this is due to the abnormalities created in different stages of gestation, implantation failure, and the impairment of the spiral vascular remodeling (8).

Reproductive system is sensitive against variety of chemical agents present in work and living environment. One of these compounds is Benzo(a)pyrene (BaP). BaP is an environmental pollutant present in fried and grilled foods, polluted climates, cigarette smoke, car emission, fume from factories, and burning forests (3, 11, 12). This compound enters the body through eating and breathing. In the previous study, toxicity-induced BaP is established (11). Although diverse studies have reported BaP-induced toxic effects on the female reproductive system, effects of BaP on endometrial receptivity is not clear and we found just one research about this. At the moment, lots of fertility-assisting techniques are applied for treating infertile couples, but despite these advances, implantation rate is relatively low. The statistics denote that the average implantation rate in every IVF is around 25% and uterus inadequate receptivity is responsible for two-thirds of the lack of these implantations, whereas the inappropriate quality of the embryos is responsible for one-third of these failures (6).

Thus, considering the environmental pollution rise and its importance in endometrial receptivity in the successful pregnancy, the present study was designed to investigate BaP${}^{{}^{'}}$s effect on histological changes in the endometrium, expression of endometrium receptivity related-genes (ALK5 and E-cadherin), implantation rate, and estrogen and progesterone levels in mice.

## 2. Materials and Methods

### Animals

Male and female BALB/c mice (25–30 gr and 8 wk) were provided by the Laboratory Animals Center of the Medical Science, University of Mazandaran. The mice were housed in the specific room at 25${}^{\circ }$C, humidity Rang of 55% and under 12 hr light/dark cycles with Free access to water and standard diet. They were kept 1 wk to acclimate to the experimental environment.

### Chemical

Benzopyrene powder (Sigma- Aldrich, China (Cat.No.B1760) was dissolved in corn oil for each group before application and used freshly.

### Experimental groups and study design

After 7 days of acclimation period, the female mice were superovulated via intraperitoneal injection with 7.5 IU pregnant mare serum gonadotropin and human chronic gonadotropin within 48 hr. Then, the female mice were mated with the male mice and examined for a vaginal plug in the next morning. The day a vaginal plug was formed was designated as zero-day of gestation (Day 0).

The pregnant mice were divided randomly into five groups of eight mice as follows: experimental groups received the doses of 100 µg/kg, 200 µg/kg, and 500 µg/kg BaP dissolved in corn oil, the control group received normal saline, and the sham group received corn oil as the vehicle of BaP. All groups received these solutions as gavage from Day 1 to Day 5 of gestation.

### Implantation site assessment

**Figure 1:**
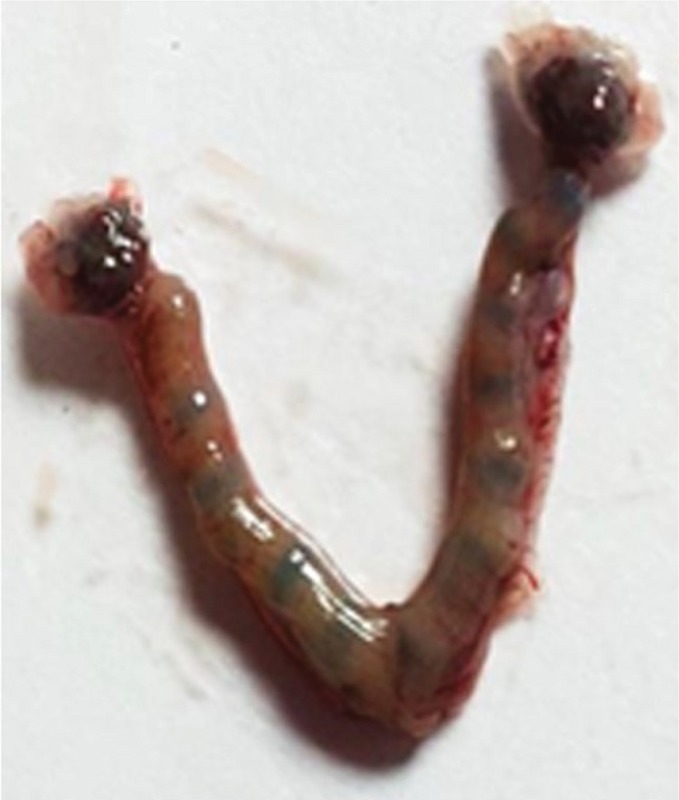
The implanted embryos seen as blue bands.

To assay the embryo implantation on Day 6 of gestation, the mice were anesthetized with 50 mg/kg Ketamine and 5 mg/kg Xylazine, and they were sacrificed after injection trypan blue (100 µL, 4%) via the tail vein (13). Then uteri were excised and examined by stereomicroscope (Nikon, Japan). The number of the implanted embryos was seen as blue bands and were counted and recorded (Figure 1).

### Hormonal assay

Immediately after the anesthetization of the animals, the blood sample was collected from their heart. Serum was harvested by centrifugation at 3000×g, 4ºC for 5 min and stored at –20ºC. The progesterone and estrogen levels were measured by using Enzyme-linked Immunosorbent Assay Kit in accordance with the manufacturer's protocol (Cat.No.E0243mo and Cat.No.E0259mo).

### Histopathological study

Mouse uterus was fixed in formalin 10%, then dehydrated and embedded in paraffin. The tissue sections were prepared at 5 μm thickness from each sample, and then stained with hematoxylin and eosin. The stained samples were assessed by light microscope and the average diameter of uterine tube, the thickness of endometrium (from the basement membrane to the apical surface of epithelial cells) and the thickness of epithelium (from the base to the apical surface of epithelial cells) were measured by Image Focus Euromex software (Japan).

Considering the fact that these indices were different in different sections of the tissue cutting, measurements were made in all samples, 10 sections of each sample and 5 fields of view of each section, and finally, the mean of them was recorded as the final number. For counting the number of glands, stromal cells and vessels, in all samples, 10 sections of each sample and in 5 fields were counted with a magnification of 40×.

### Real-time polymerase chain reaction (RT-PCR)

Total RNA was extracted from the mouse endometrial tissue (Qiagen RNeasy plus mini kit.cat.nos.74134, 74136). The integrity of total RNA was assessed by agarose gel. RNA was quantified by measuring optical density at 260 nm. The extracted RNA was reverse-transcribed to cDNA using cDNA synthesis kit from BIONEER Company (AccuPower CycleScript RT PreMix dN6). The real-time PCR reactions consisted of 10 μl SYBR Premix Ex Taq tm II 2x (Tli RNaseH plus, Takara, cat.RR820Q), 1.5 μl cDNA from each sample, 0.5 μl from each Reverse and Forward primers (primers' sequences as shown in Table I), 7.5 μl water and total volume 20 μl.

The real-time condition for reactions was as follows: 95 for 30 sec, 95 for 15 sec, and 60 for 30 sec, and the cycles were repeated 40 times, melting curve from 95 to 65 with 0.5 every 5 sec. Relative gene expression was calculated by pfaff formula (14) and Gene Ex software. Comparison between the groups was analyzed by the LSD method with the minimum meaningful difference.

### Ethical consideration

All the experimental methods were manipulated by the Institutional Animal Ethics Committee of the Mazandaran University Medical Sciences (IR.MAZMUS.REC. 1395.2507).

### Statistical analysis

Values were expressed as the mean ± standard deviation (SD). Collected data were analyzed using ANOVA and Tukey's test for comparison between the groups. Graph pad prism software was used for statistical analyses and *p* < 0.05 was considered significant.

## 3. Results

### Implantation sites finding

**Figure 2:**
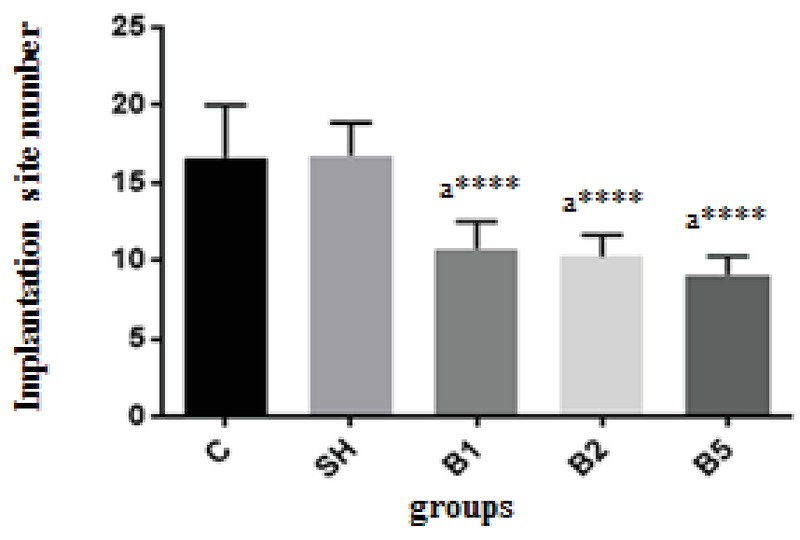
BaP decreased the number of implantation sites in mice (*n* = 8) C: Control group receiving normal saline, SH: Sham group receiving corn oil, B1: The group receiving BaP as 100 µg/kg, B2: The group receiving BaP as 200 µg/kg, B5: The group receiving BaP as 500 µg/kg. The values are as mean ± SD. a: Comparing diverse Benzopyrene-receiving groups with the control group (*p* < 0.0001:****).

Implantation site findings are presented in Figure 2. The number of implantation sites significantly decreased in all BaP-receiving groups (B1: 10.75 ± 1.75, B2: 10.25 ± 1.39, B5: 9.13 ± 1.13) compared with the control (16.63 ± 3.38) and sham (16.75 ± 2.12) groups (*P* < 0.0001). But no significant difference was observed between the different doses of BaP in the number of implanted embryos.

### The serum levels of estrogen and progesterone

**Figure 3:**
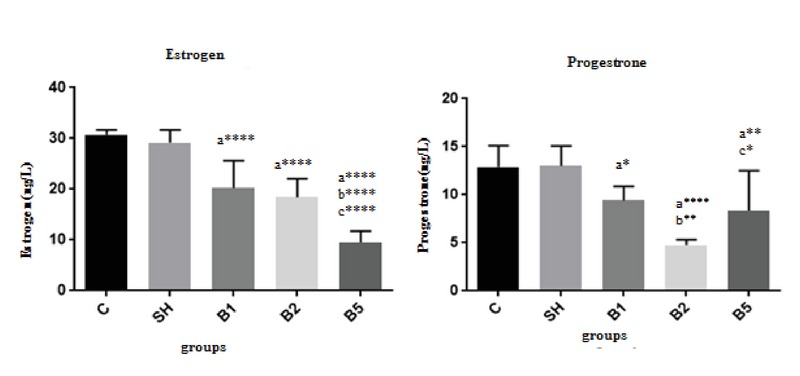
BaP's effect on estrogen and progesterone levels in the pregnant mice (n = 8). C: The control group received normal saline, SH: Sham group receiving corn oil, B1: The group receiving BaP as 100 µg/kg, B2: The group receiving BaP as 200 µg/kg, B5: The group receiving BaP as 500 µg/kg. The values are as mean ± SD (p < 0.0001:****, p < 0.01:**, p < 0.05:*). a: Comparing diverse BaP-receiving groups with the control group, b: Comparing with B1, and c: Comparing with B2; C: The control group received normal saline; SH: The Sham group receiving corn oil; B1: The group receiving BaP as 100 µg/kg; B2: The group receiving BaP as 200 µg/kg; B5: The group receiving BaP as 500 µg/kg.

Serum estrogen and progesterone levels are presented in Figure 3. The level of estrogen significantly decreased in all BaP-receiving groups (B1: 20 ± 5.37 ng/l, B2: 18.4 ± 3.64ng/l, B5: 9.53 ± 2.25 ng/l) compared with the control (30.75 ± 0.89 ng/l) and sham (29.19 ± 2.49 ng/l) groups (*P* < 0.0001). Also, progesterone level at different concentrations (B1: 9.41 ± 1.46 ng/l, B2: 4.78 ± 0.56 ng/l, B5: 8.36 ± 4.15 ng/l) of BaP significantly declined when compared with control (12.88 ± 2.33 ng/l) group (*p*:0.0496, *p* < 0.0001 and *p* = 0.0054, respectively).

### Histological finding

**Figure 4:**
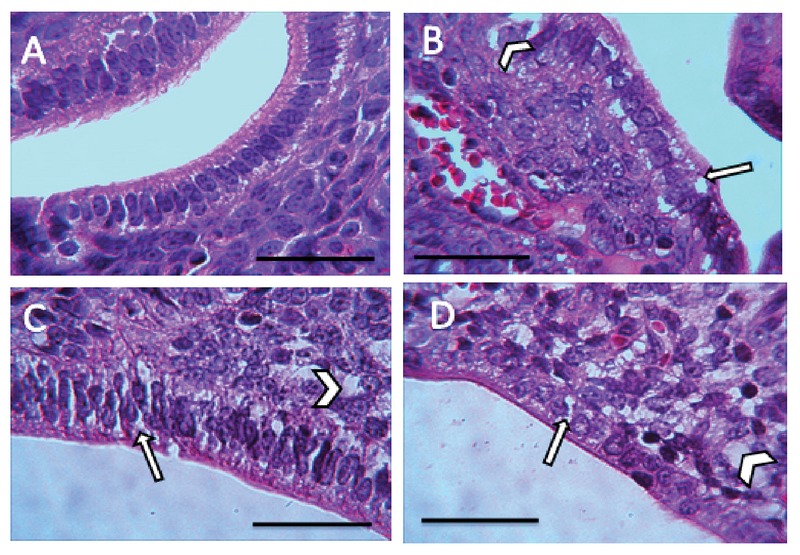
The presence of vacuole in stromal cells (arrowhead) and epithelial cells (arrow) (H& E staining, magnification ×100) A: the control group receiving normal saline, B: the group receiving BaP as 100 µg/kg, C: the group receiving BaP as 200 µg/kg, D: the group receiving BaP as 500 µg/kg. Scale bar = 150µ.

**Figure 5:**
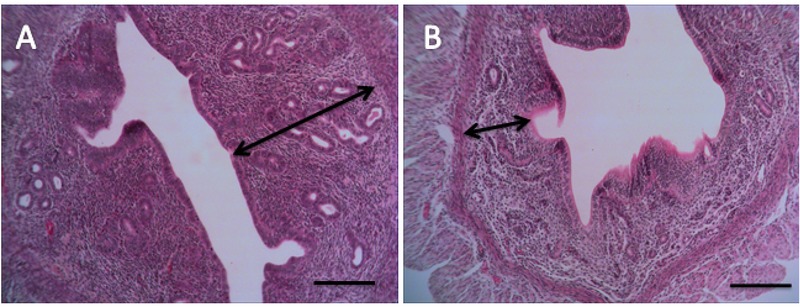
Reduction of endometrial thickness in the BaP group (B) compared to the control group (A), (H& E staining, magnification ×10), Scale bar = 150µ.

**Figure 6:**
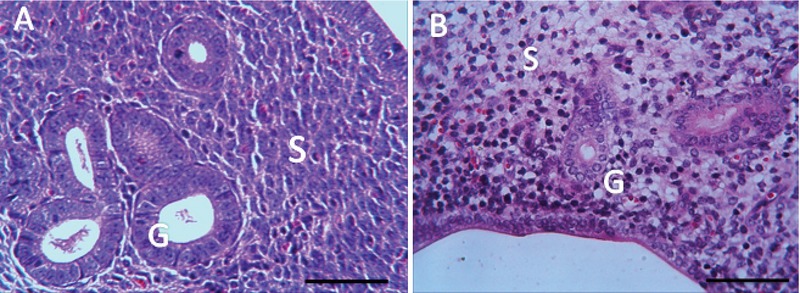
BaP reduced the number of stromal cells and endometrial glands/cross-sections A: The control group receiving normal saline, B: The group receiving BaP as 500 µg/kg. G: Gland, S: Stroma, (H& E staining, magnification ×40), Scale bar = 150µ.

**Figure 7:**
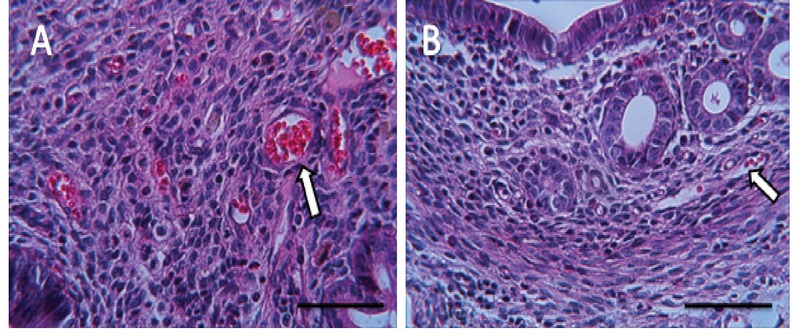
BaP reduced the number of blood vessels. A: The control group received normal saline, B: The group receiving BaP as 500 µg/kg (H& E staining, magnification ×40), Scale bar = 150µ.

The microscopic findings showed that BaP reduces the number of the implantation site. To examine the microscopic structure, endometrial morphology was evaluated by H&E staining. BaP reduced epithelium and endometrium thickness when compared with the control group. The number of stromal cells and endometrial glands/cross-sections were significantly different between control and BaP groups. Control group had a closed uterine lumen, while the lumen's diameter increased in BaP groups. The mouse uteri in the BaP groups also showed a decrease in the number of blood vessels in the endometrium compared with control group (Table II). Vacuole was observed in epithelial and stromal cells in the BaP groups. These results indicate that BaP exerts destructive effects on the endometrial morphology in doses of 100, 200, and 500 µg/kg (Figures 4–7).

### Effect of BaP on ALK5 and E-cadherin genes expression level

**Figure 8:**
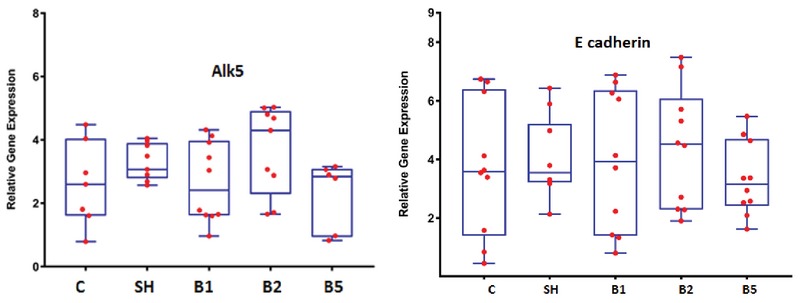
BaP's effect on ALK5 and E cadherin Genes Expression Level in the pregnant mice (*n* = 8). C: The control group received normal saline, SH: Sham group receiving corn oil, B1: The group receiving BaP as 100 µg/kg, B2: The group receiving BaP as 200 µg/kg, B5: The group receiving BaP as 500 µg/kg. The values are as mean ± SD.

To assess BaP molecular effects on implantation, ALK5 and E-cadherin genes expression level was analyzed. The Real-Time RT- PCR results indicate that these two genes expression level in BaP groups had not revealed any significant difference compared with the control group (Figure 8).

**Table 1:** Primers' sequences for Activin receptor like kinase 5(ALK5), E-cadherin and B-actin.


ALK5	Forward	GATTTATAGCAGCAGACAACA
	Reverse	TCATTCCTTCCACAGTAACA
E cadherin	Forward	AACGCTCCTGTCTTCAAC
	Reverse	GCATCATCATCGGTCACT
β actin	Forward	GCCTTCCTTCCTGGGTAT
	Reverse	GATCTTGATCTTCATGGTGC

**Table 2:** BaP's effect on endometrial morphology in the pregnant mice (*n *= 8).


**Groups**	**Endometrial Epithelium (µm)**	**Lumen (µm)**	Gland (N/HPF)*	Vessel (N/HPF)*	**Endometrial Diameter (µm)**	Stromal Cell (N/HPF)*
C	24.4 ± 5.01	137.1 ± 13.99	10.96 ± 2.43	14.14 ± 2.74	609.7 ± 137.5	134.9 ± 11.97
SH	21.1 ± 2.81	122.2 ± 28.84	10.13 ± 1.65	12.76 ± 1.61	553.6 ± 143.4	133.4 ± 25.92
B1	18.86 ± 1.40${}^{a}$	173 ± 40.11	7.33 ± 1.99${}^{a}$	7.18 ± 1.48${}^{a}$	417.2 ± 95.3${}^{a}$	96.25 ± 18.71${}^{a}$
B2	15.11 ± 4.07${}^{a}$	213.9 ± 40.63${}^{a}$	6.77 ± 1.71${}^{a}$	6.93 ± 1.53${}^{a}$	320.4 ± 69.87${}^{a}$	96.13 ± 11.41${}^{a}$
B5	13.7 ± 1.09${}^{ab}$	280.4 ± 52.34${}^{abc}$	7.53 ± 2.24${}^{a}$	7.49 ± 2.28${}^{a}$	439.2 ± 35.18${}^{a}$	87.97 ± 12.82${}^{a}$

Note: The values are as mean ± SD (*p* < 0.05);

*: Number of cross-sections per high power field (HPF);

a: Comparing diverse BaP-receiving groups with the control group, b: Comparing B5 and B2 with B1, and c: B5 with B2;

C: The control group received normal saline;

SH: The Sham group receiving corn oil;

B1: The group receiving BaP as 100 µg/kg;

B2: The group receiving BaP as 200 µg/kg;

B5: The group receiving BaP as 500 µg/kg.

## 4. Discussion

Although in recent years, various studies reported the harmful effects of BaP on female reproduction system (11, 15), very little attention has been given to the effects of BaP on embryo implantation and endometrial receptivity. In this study, findings show that BaP decreased the number of implantation sites, serum levels of estrogen and progesterone, and changes endometrial morphology, but no significant difference was observed in ALK5 and E-cadherin genes expression level.

In this study, estrogen and progesterone were reduced in all BaP-receiving groups compared with the control group. These results have also been obtained in the research by Anthony E Archibong co-worker, in which the pregnant rats were exposed to BaP from D10 to D20 of gestation (11). These researchers in another study (2012) showed that BaP has harmful effects on ovarian function. In that study, The female rats were exposed to this compound in the period before mating and during menstruation cycle, in which the results show that BaP leads to the reduction of estrogen and progesterone hormones (15). Archibong and colleagues reported phenanthrenequinone, a metabolite of BaP family, that is able to inhibit progesterone secretion from luteal cells by in vitro (11). Only one study reported estrogen and progesterone level increase in the pregnant mice exposed to BaP (3).

Our study shows that BaP leads to compromised embryo implantation that is a critical stage of pregnancy. So, the number of the implantation sites was reduced in all BaP groups compared to the control group. Our findings are consistent with those by Zhao and colleagues. Estrogen and progesterone are the key factors for implantation and pregnancy maintenance in all animal species (3). This decrease in implantation site seems to be due to the reduction of these hormones.

In this study, we investigated some morphological factors contributing to the endometrial receptivity such as endometrial thickness, uterine epithelial thickness, endometrial glands number, uterine lumen diameter, endometrial blood vessel, stromal cells number and vacuolization. Uterine lumen diameter was increased in mice of the 200 µg/kg and 500 µg/kg BaP groups compared with the control group. Zhao and colleagues in their study examined some of these histological factors, and their results were consistent with our results (3). Endometrial morphology plays a critical role in successful implantation. During the implantation window under the influence of estrogen and progesterone, much significant morphological changes occur in the uterus that is necessary for endometrial receptivity and embryo implantation (6). It seems that BaP with the decrease of estrogen and progesterone can change endometrial morphology. Closure of the Lumen helps the better adhesion of embryo to the uterine epithelium (16). Pinopods, cytoplasmic projections from the uterine epithelium, are observed as the adequate Progesterone level and just at the time of implantation window. Pin pods pinocytosis intraluminal fluid into the stroma and increases endometrial edema. This edema leads to luminal closure (16, 17). Therefore, luminal closure during the implantation mainly depends on progesterone. Previous studies also show that luminal closure doesn't occur in the absence of progesterone (8, 18).

It seems that the decreased luminal closure in different BaP-receiving groups can be due to the progesterone level reduction. The development and formation of the vascular network in the endometrium are one of the major morphological transformations during the pregnancy. In this study, the reduction of blood vessels is seen in all experimental groups compared with the control group. This endometrial factor hasn't been analyzed in the previous studies. During the implantation and early post-implantation period, the decidual transformation is associated with the formation of new vessels and anastomosis network connecting maternal and embryonic tissue (19).

This vascular network is responsible for exchanging respiratory gases, nutrient, and wastes required for embryo development (20). Studies denote that insufficient growth of the vessels reduces the normal chance of implantation (21). So far, various molecular factors have been identified playing an important role in vascularization, the most critical of which is vascular endothelial growth factor (VEGF). Studies show that VEGF expression is hormonal regulated (19). Some researcher demonstrated that the signaling pathway of VEGF-progesterone is a key regulator for decidual angiogenesis so that the damage to this signaling pathway can cause placenta failure, disturbance of blood supply, and abortion (22). Blood-vessel reduction in the BaP groups can be the case by estrogen and progesterone drops and/or as a result of the oxidative stress induced by BaP. In this study, the number of endometrial glands was analyzed. The results show that glands' number in the endometrium declined in all BaP-receiving groups. These findings are consistent with those by Zhao and co-worker (3).

Endometrial glands are a source of nutrient and secretion of growth factors, adhesion molecules, and immune system factors. The secretion of glands includes carbohydrates, protein, and fat as a significant way for food exchange in early pregnancy before the placenta is established. In addition, glands secrete the growth factors and cytokine, playing a significant role in successful pregnancy. Studies indicate that lack of the glands' activity and their number and secretions decrease results in pregnancy failure (23). According to the studies, estrogen increases the number of glands and change the type of glandular epithelia (24). While progesterone leads to the glands' enlargement and active secretion (25). Thus, the reduction of estrogen and progesterone level in the BaP groups can be the reason behind the glands' number reduction in this study. Endometrial thickness is an important factor in successful implantation. Several studies reported that a thin endometrium is associated with implantation failure (26). In the present study, the reduction of endometrium thickness observed in all BaP-receiving groups was compared with the control group, which hasn't been analyzed in the previous studies. Studies demonstrated that embryo does not develop well in the environments with high oxygen tension as a result of the oxidative stress increase. When endometrium thickness is less than the normal range, the functional layer is thin, and so embryo can be closer to the spiral vessels and higher oxygen concentration because oxygen concentration in the basal layer is more than the functional layer. Moreover, there are evidence suggesting that the secretion of cytokines is low in the women with thin endometrium (27). Proliferation and differentiation of endometrial stromal cells is also essential for implantation and decidualization (28).

Paracrine factors secreted by these cells play a critical role in setting the maternal immune system, remodeling of the uterus, angiogenesis, and early embryonic growth (29). In this study, the number of stromal cells decreased in all experimental groups in comparison with the control group. Our results match with those by Zhao and colleagues (3). Epithelial and stromal cell proliferation occur under the control of the estrogen and progesterone (30). Also, at the time of decidualization, the cytokines production rise brings about the migration of immune cells to the endometrium (31). Studies suggest that estrogen plays a role in the infiltration of immune cells to the endometrium (28). Therefore, it seems that the reduction of stromal cells in BaP-receiving groups can be due to dropped cell proliferation and also reduced immune cells infiltration as a result of estrogen and progesterone levels change. Cell Vacuolization is a cell degeneration morphological sign occurring after the tissues' exposure to bacterial or viral pathogens, industrial compounds, ischemic injury, cell manipulation, pharmacological agents, and toxic elements (32, 33).

In the present research, vacuolization was observed in epithelial and stromal cells. The formation of vacuoles in cell cytoplasm under the influence of diverse chemical compounds like the industrial pollutions has been reported in many studies (32). During the uterine receptivity, the endometrial epithelium cells proliferate for the embryo attachment to the endometrium (28). The endometrial epithelium cells proliferation is a prerequisite for implantation. The normal function of luminal epithelium and its secretions are critical for the embryo attachment and implantation and play a critical role in pregnancy maintenance (34). In our study, the decrease of epithelium cell height was found, which hasn't been analyzed in the previous studies. Estrogen increase cell proliferation and change in epithelial cells morphology from cuboidal into columnar secretory. The subsequent increase in cell count (hyperplasia) and cell size (hypertrophy) lead to increase in the epithelial cells' height (28). Although few studies have reported that the severe decrease in progesterone level can decrease endometrial epithelial cell proliferation and cell apoptosis rise (35), epithelial height drop in BaP-receiving groups can be due to reduced epithelial cells' number and size created by lower estrogen level.

In our study, ALK5 and E-cadherin genes expression level were analyzed and no meaningful difference was found in mice-treated BaP compared with the control group. Although Zhao and co-workers reported that E-cadherin gene expression level increases with BaP exposure (3), the reason for this difference was related to the dose of BaP. We chose BaP in this study based on the dose of this substance in the nature and diet of individuals. The effect of BaP on ALK5 gene expression level hasn't been addressed in the previous studies. It seems that BaP's effect on endometrial receptivity and implantation can be due to the hormonal and morphological transformation of the endometrium. Implantation-related genes expression in mouse reduces on D6 of gestation, and uterus epithelial cells lose the appropriate condition for attachment reaction. Studies show that blastocyst can initiate implantation beyond the normal window of implantation under appropriate progesterone level because progesterone partially improves these genes' expression, and this indicates that progesterone level decline is a critical limiting factor in uterus receptivity (2). Our study showed that although BaP did not affect the expression of these genes with this dose, reduction in the implantation site may be due to the decrease of progesterone hormone.

## 5. Conclusion

The results here show that BaP can exert detrimental effects on successful pregnancy in the early stages, and due to the change in endometrial morphology and estrogen and progesterone level, endometrial receptivity and implantation can be damaged in mice.

##  Conflict of Interest

There is no conflict of interest in this study and publication.
